# Multi-objective cluster based bidding algorithm for E-commerce search engine marketing system

**DOI:** 10.3389/fdata.2022.966982

**Published:** 2022-09-26

**Authors:** Cheng Jie, Zigeng Wang, Da Xu, Wei Shen

**Affiliations:** Walmart Labs, Sunnyvale, CA, United States

**Keywords:** clustering, intention embedding, SEM bidding, multi-objective, optimization

## Abstract

Search engine marketing (SEM) is an important channel for the success of e-commerce. With the increasing scale of catalog items, designing an efficient modern industrial-level bidding system usually requires overcoming the following hurdles: 1. the relevant bidding features are of high sparsity, preventing an accurate prediction of the performances of many ads. 2. the large volume of bidding requests induces a significant computation burden to offline and online serving. In this article, we introduce an end-to-end structure of a multi-objective bidding system for search engine marketing for Walmart e-commerce, which successfully handles tens of millions of bids each day. The system deals with multiple business demands by constructing an optimization model targeting a mixture of metrics. Moreover, the system extracts the vector representations of ads *via* the Transformer model. It leverages their geometric relation to building collaborative bidding predictions *via* clustering to address performance features' sparsity issues. We provide theoretical and numerical analyzes to discuss how we find the proposed system as a production-efficient solution.

## 1. Introduction

In this article, we consider the problem of building an industrial-level scale *search engine marketing* (SEM) system aiming at promoting the company's business by showing and recommending advertisements on search-result pages. Among various forms of online advertising, *sponsored search auctions* often contribute significantly to online advertising revenue as search results often have more prominent exposure.

Since the onset of search engines such as Google and Yahoo, designing an efficient SEM model is continuously attracting attention from both academia and industry, and the emerging challenges appeal particularly to the co-domain of economics and computer science. Over the years, a large body of literature has studied the constrained bidding optimization model, which maximizes business objectives under the prefixed spending limit. For instance, Borgs et al. (2007) and Feldman et al. (2007) establish SEM bidding models for a single advertiser as constrained optimization problems in a deterministic setting where the advertisers' position, clicks, and the cost associated with a bid are known *a priori*. In comparison, SEM bidding as an optimization problem under the stochastic setting has been studied in Pin and Key (2011) and Abhishek and Hosanagar (2012). Game-theoretic structures of SEM have been studied by Borgers et al. (2013) and Aggarwal et al. (2009), and both studies aim to boost the welfare of all advertisers on search engine platforms. More recently, a new stream of work has emerged which formulate the SEM bidding optimization as a dynamic pricing problem (Cheng, 2018) by incorporating the sequential behavior of SEM ads (Dayanik and Parlar, 2013; Shen et al., 2020).

In practice, however, we found that the optimization SEM bidding models built by the seminal works are often too restrictive for practical implementation, although they are usually rigorously justified through mathematical theories. In particular, the high volume of candidate ads is a crucial factor that hurdles the applicability of those methods in the real world. We must tackle two main challenges when dealing with the industrial scale of candidate ads. First, due to the limited number of search-engine platform ad slots, most SEM ads' feedback data are inevitably sparse, preventing an accurate and effective estimation of their performances. Second, under the high volume of ads, bidding evaluations through complex optimization algorithms are too costly to implement, especially when we demand a high frequency of bidding operations.

To address the above challenges induced by the high volume of ads, we introduce a generic bidding framework that is currently in production for the multi-million-scale ads bidding for Walmart's e-commerce business. The solution of our system comprises two major components:

- A deep-learning-based multi-stage predictive algorithm for predicting the performance of the advertisement through their multi-modality signals, including the user feedback data and the contextual features of ads;
- A multi-optimization algorithm that assigns a bidding price for each ad based on its performance forecast according to the objectives of business demands.

Toward building such a system, we first construct a language model to extract vector representations of the ads through deep-learning Transformer (Vaswani et al., 2017) architectures. After capturing the customer's intention of the ads page through vector representations, we can now fully leverage the geometric characteristics of the representations to aggregate ads' information that would be sparse otherwise. The multi-stage prediction algorithm then enriches the grouping patterns of features *via* ads clustering, further alleviating the sparsity issue of the features. In the meantime, the clustering-based solution improves the scalability of the second-stage optimization algorithm by significantly reducing the number of entities in the downstream evaluation of the bids.

### Our contributions^1^

Overall, the contributions of this study are summarized as follows:

- We are the first to propose an end-to-end multi-objective SEM bidding framework that incorporates deep learning-based ads representation, clustering, and prediction.
- We formulate a multi-objective bidding optimization problem and theoretically justify a proposed solution toward the optimization problem. The solution is used to evaluate each SEM ad's bidding price.
- To generate vector representations of SEM ads, we present a novel approach utilizing historical user-item level engagement data and Transformer architectures.
- To cluster large-scale SEM ads based on their embedding vectors, we introduce a multi-stage method that significantly reduces the computation cost.
- We use numerical analysis to reason the theoretical assumptions of our multi-objective bidding model and conduct offline and online experiments to illustrate the significant benefits of our proposed bidding system.

*Related work*. The previous literature addresses the sparsity issue primarily by using the ads' “keywords” in addition to the feedback data (Hillard et al., 2010). However, using word tokens as a categorical feature can pose severe problems in building predictive models due to the high cardinality. Unlike (Hillard et al., 2010), our approach constructs continuous vector representations of ads and, therefore, avoids the tenuous work of dealing with massive word tokens. We point out that the idea of clustering SEM ads have also been proposed to overcome the high computation demands (Mahdian and Wang, 2009; Chen et al., 2013). However, the clustering algorithms developed in the above study are based on the distributions of SEM ads' historical feedback data, thereby excluding those with sparse historical features, which is problematic for modern SEM applications.

The rest of the article is organized as follows: In Section 2, we introduce the mathematical formulation of the SEM bidding model and present an overview of the infrastructure of the SEM bidding system. Section 3 illustrates the details of the SEM ad embedding methods and the proceeding two-stage clustering algorithm. With SEM ads clusters being established, Section 4 layout the model training process of predicting the performance metrics of SEM ads. In Section 5, we thoroughly examine the performance of the proposed cluster-based bidding solution *via* both offline studies and online experiments. As expected, the clustering step is essential for trading off the sparsity, accuracy, and scalability.

## 2. Background for SEM bidding

We first introduce the underlying bidding model and system that power Walmart's SEM business.

### 2.1. Multi-objective SEM bidding model

In real-world practice, an E-Commerce company usually tends to use marketing dollars to drive multiple business goals in order to achieve balanced performance. Common business objectives include but are not limited to revenue-related targets such as gross merchandise value (GMV) and consumption profits and customer exposure targets such as clicks and acquisitions. Different business objectives are inevitably not aligned with each other. Therefore, a mixture of business goals brings about ambiguity in defining and modeling the problem. To address such an issue, we establish our SEM bidding model as an optimization problem aiming at maximizing a weighted sum of various business objectives.

### Notations and optimization problem formulation

To better formulate the multi-objective model, we refer to each business objective *i* involved in the problem as a reward *i*, denoted as *R*_*i*_. Moreover, since we construct the SEM bidding template *via ad groups* generated by the procedures described in Section 3, the reward *i* associated with an adgroup *g* is, therefore, identified as *R*_*ig*_. Suppose an advertiser aims to maximize a weighted sum of a set I of rewards given budget *B*, the SEM bidding can be given by


(1) $$\begin{array}{l} \underset{\left\{b_{g}\right\}}{\max}E\left[\underset{g\in G}{\sum }\underset{i\in I}{\sum }w_{i}R_{ig}\left(b_{g}\right)\right] \end{array}$$

(2) $$\begin{array}{l} \text{s.t.}E\left[\underset{g\in G}{\sum }S_{g}\left(b_{g}\right)\right]\leq B, \end{array}$$

where *b*_*g*_ is the bidding value assigned to the ads at group *g*, *R*_*ig*_(·) and *S*_*g*_(·) are the corresponding *reward* and *spend* functions. Meanwhile, *w*_*i*_ represents the weight of each reward in the optimization objective.

Directly solving (1) is impractical since the expected rewards *E*(*R*_*ig*_(*b*_*g*_)) and expected spending *E*(*S*_*g*_(*b*_*g*_)) can be very complicated (Feldman et al., 2007). However, by adding certain practical assumptions on *E*(*R*_*ig*_(*b*_*g*_)) and *E*(*S*_*g*_(*b*_*g*_)), the optimum of (1) can be found quite efficiently. To this end, we first denote the *expected click* for the bid value of *b* as *E*[*C*_*g*_(*b*)], and introduce the notions of RPS (*reward per spend*) and RPC (*reward per click*) below.

#### 2.1.1. Definition 1

For each reward *R*_*i*_, The *RPS*_*i*_, i.e, reward per spend (revenue of an ad per unit of spend), equals: $RPS_{ig}=\frac{E\left[R_{ig}\left(b_{g}\right)\right]}{E\left[S_{g}\left(b_{g}\right)\right]}$ given an ad group *g*. The *RPC*, i.e, reward per click, equals $RPC_{ig}=\frac{E\left[R_{ig}\left(b_{g}\right)\right]}{E\left[C_{g}\left(b_{g}\right)\right]}$ for a given ad *g*.

We now state the critical assumption.

#### 2.1.2. Assumption 1

For a given ad group *g*, its reward per click *RPC*_*ig*_ is invariant to the change of bid value *b*_*g*_. Furthermore, we suppose *E*[*C*_*g*_(*b*_*g*_)] = *c*_*g*_ · *b*_*g*_ for a given constant of *c*_*g*_. When the search engine uses the first-price auction^2^, we have $E\left[S_{g}\left(b_{g}\right)\right]=c_{g}\cdot b_{g}^{2}$ as a result.

Under Assumption 2.1.2, we have the following key result:

#### 2.1.3. Theorem 1

The optimal solution to the optimization problem in (1) is achieved when the weighted sum of RPS (reward per spend) is the same for all g∈G.                □

*Proof*. The Lagrangian of (1) is given by:


(3) $$\begin{array}{l} L=E\left[\underset{g\in G}{\sum }\underset{i\in I}{\sum }w_{i}R_{ig}\left(b_{g}\right)\right]-\lambda \left\{B-E\left[\underset{g\in G}{\sum }S_{g}\left(b_{g}\right)\right]\right\}, \end{array}$$

The KKT condition for the gradient of (1) is:


$$\begin{array}{l} \forall g:\frac{dL}{db_{g}}=\frac{d}{db_{g}}E\left[\underset{i\in I}{\sum }w_{i}R_{ig}\left(b_{g}\right)\right]-\lambda \frac{d}{db_{g}}E\left[S_{g}\left(b_{g}\right)\right]=\\ 0,\lambda >=0 \end{array}$$

Since ∀*i*, *R*_*ig*_(*b*_*g*_), and *S*_*g*_(*b*_*g*_) are independent of other ad groups. KKT condition of (3) implies that an optimal solution exists when:


$$\begin{array}{l} \frac{d}{db_{g}}E\left[\underset{i\in I}{\sum }w_{i}R_{ig}\left(b_{g}\right)\right]/\frac{d}{db_{g}}E\left[S_{g}\left(b_{g}\right)\right] \end{array}$$

takes the same value across g∈G. Under assumption 2.1.2, we immediately have, for ∀*i*,


$$\begin{array}{l} E\left[R_{ig}\left(b_{g}\right)\right]=c_{g}b_{g}RPC_{ig},\\ E\left[S_{g}\left(b_{g}\right)\right]=c_{g}b_{g}^{2} \end{array}$$

(4) $$\begin{array}{l} \frac{d}{db_{g}}E\left[R_{ig}\left(b_{g}\right)\right]/\frac{d}{db_{g}}E\left[S_{g}\left(b_{g}\right)\right]=\frac{RPC_{ig}}{2b_{g}}. \end{array}$$

As a result, the weighted sum of rewards satisfies the following


$$\begin{array}{l} \frac{d}{db_{g}}E\left[\underset{i\in I}{\sum }w_{i}R_{ig}\left(b_{g}\right)\right]/\frac{d}{db_{g}}E\left[S_{g}\left(b_{g}\right)\right]\\ =\frac{\sum_{i\in I}w_{i}c_{g}RPC_{ig}}{2c_{g}b_{g}}=\frac{\sum_{i\in I}w_{i}RPC_{ig}}{2b_{g}} \end{array}$$

Moreover, for ∀*i*, the reward per spend is: $RPS_{ig}=\frac{E\left[R_{ig}\left(b_{g}\right)\right]}{E\left[C_{g}\left(b_{g}\right)\right]}=\frac{RPC_{ig}}{b_{g}}$, and the weighted sum of reward per spend equals


$$\begin{array}{l} \underset{i\in I}{\sum }w_{i}RPS_{ig}=\frac{E\left[\sum_{i\in I}w_{i}R_{ig}\left(b_{g}\right)\right]}{E\left[C_{g}\left(b_{g}\right)\right]}=\frac{\sum_{i\in I}w_{i}RPC_{ig}}{b_{g}} \end{array}$$

Hence, the KKT condition of (3) is equivalent to saying that the weighted sum of $\underset{i\in I}{\sum }w_{i}RPS_{ig}$ are equal across g∈G.

Recall that Assumption 2.1.2 claims that *RPC*_*ig*_ is steady against *b*_*g*_. Therefore, Theorem 2.1.3 implies that as long as we have an accurate prediction of *RPC*_*ig*_ for each group and each type of reward *R*_*i*_, the optimal condition in (1) can be easily achieved by setting the bids *b*_*g*_ such that $\underset{i\in I}{\sum }w_{i}RPC_{ig}/b_{g}$ are equal across all ad groups *g*.

#### 2.1.4. Remark 1

Note that the classical singular-ad bidding algorithm can be easily recovered by replacing the ad group *g* with the single ad.

#### 2.1.5. Remark 2

As shown in Section 5, the bidding strategy derived from Theorem 2.1.3 can also retrieve a good approximation of optimality for the SEM problem (1) under second price auction^3^ in that linear relations implied from Assumption 2.1.2 still holds statistically.

### 2.2. SEM bidding system

**Figure 1:**
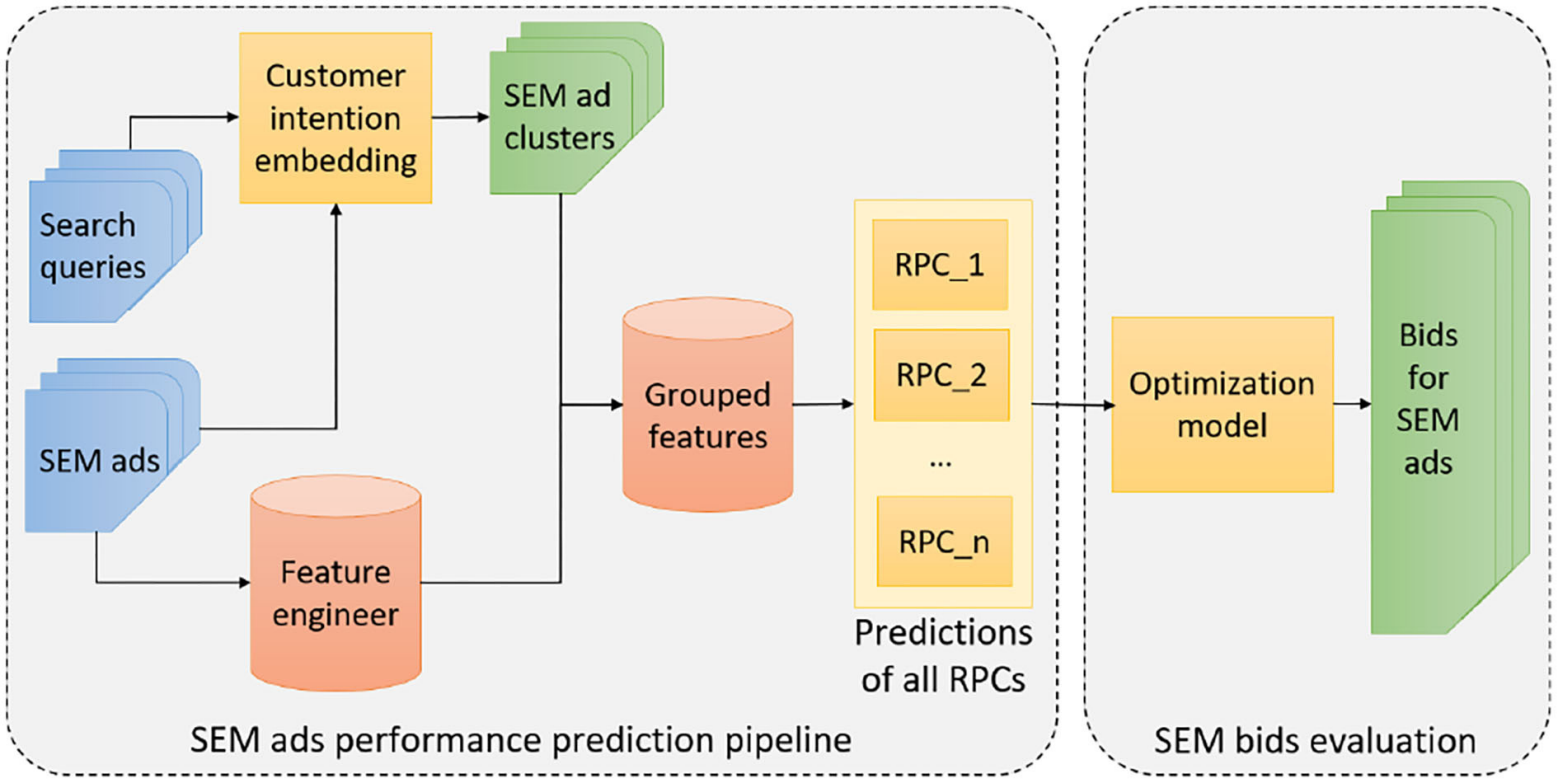
Overview of the infrastructure for providing search engine marketing (SEM) ads' bids by our approach.

The results in the previous section suggest that the critical task for determining the bids of SEM ads is to accurately predict each reward per click (RPC) for each ad group *g*. In the sequel, we propose a design of the SEM ads bidding system illustrated in Figure 1. In Figure 1, the first task for obtaining the reward per click (*RPC*_*i*_) predictions for each type of reward *i* is clustering the pool of SEM ads into ad groups. It consists of two steps: 1. building a representation learning model that encodes SEM ads into embeddings; 2. clustering SEM ads into ad groups.

After creating the SEM ad groups, the system will aggregate the features for ads within each ad group, and then train a predictive model to accurately forecast the *RPC*_*ig*_ for each ad group. We plug the *RPC*_*ig*_ back to the optimization problem and obtain the final bidding *b*_*g*_ for each g∈G as $b_{g}=\left(\underset{i\in I}{\sum }w_{i}RPC_{ig}\right)/RPS_{g}$, where *RPS*_*g*_ is known in advance. Notice that the model training process is conducted offline while the model prediction process is performed online with batch.

## 3. Embedding and clustering of SEM ads

The ad-group level bidding in (1) performs the best when each ad cluster is dedicated to a specific user intention. For this purpose, we segment the SEM ads into mutually exclusive ad clusters in terms of customer intention in two steps. First, We build the customer-intention representation model which provides an embedding for each ad. Second, based on the embeddings, we develop a multi-stage clustering method that groups the massive ads into small to mid-sized groups.

### 3.1. Customer intention embedding model

The customer intention of an SEM ad is defined as the integrated purchase intention (of the set of search queries) that leads to the clicked ads on the search engine. For example, an ad may appeal to customers who search for “apple phone 8 case” or “iPhone 9 case,” if their intentions are the case covers for various versions of the iPhone. If two ads share a large portion of clicked search queries, their customer intentions should be close to each other. Therefore, we design the customer intention model to reflect the co-click relations among the SEM ads. We propose the following metric to capture such intention.

#### 3.1.1. Interactive metric

The interactive metric (*I*) is designed to calibrate the similarity between customer intentions of two SEM ads. Given two SEM ads A1 and A2, we first obtain the numbers of co-clicks of the two ads and denote them as *C*_(*A*1*coA*2)_ and *C*_(*A*2*coA*1)_. Given the numbers of total historical clicks of the two ads *C*_*A*1_ and *C*_*A*2_, the metric value for A1 and A2 is defined *via*:


(5) $$\begin{array}{l} I_{A1,A2}=\sqrt{\frac{C_{\left(A1coA2\right)}*C_{\left(A2coA1\right)}}{C_{A1}*C_{A2}}} \end{array}$$

**Figure 2:**
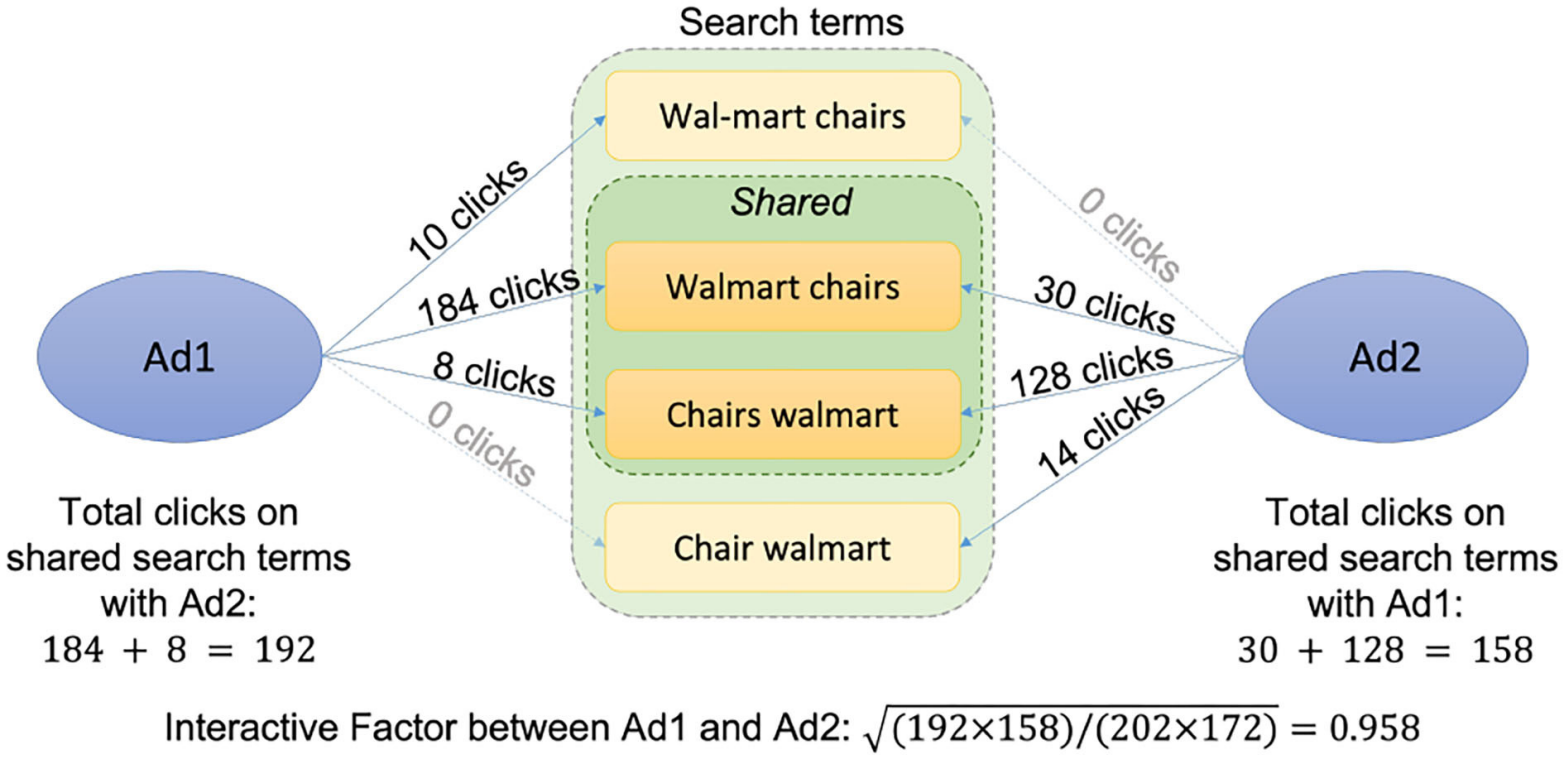
Interactive metric: An example.

Refer to Figure 2 for an illustration of interactive metric in a real-world example, which effectively discounts the popularity and exposure bias.

#### 3.1.2. Contextual features of SEM ad

When a search query appears, the search engine will try to match it with the SEM ads according to the content of their landing pages. In light of that, we select the ads' website's text content as the main feature for the customer intention model, since the content should be a critical factor in customers' decision making. The text feature of an SEM ad is a combination of titles and descriptions of products contained in the ad's website. For the SEM ads with more than one product, we choose the three top products to constrain the length of the input feature. Once the features are extracted, they are processed and converted through the standard tokenization and padding procedures described in Devlin et al. (2019).

#### 3.1.3. Transformer-based customer intention representation model

**Figure 3:**
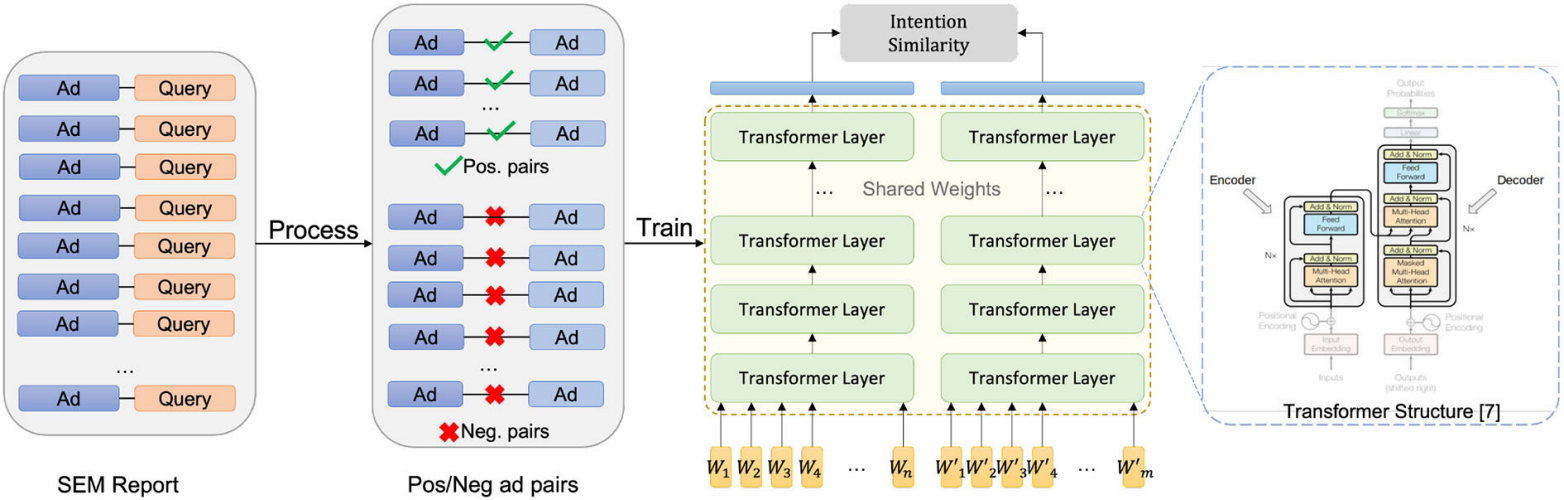
Search engine marketing ads customer intention embedding model and its training process.

Recently, the attention-based encode-decode structure transformer has become the status quo architecture for natural language processing tasks (Vaswani et al., 2017). Motivated by the structure of the bidirectional transformer from Devlin et al. (2019), we built a transformer-based deep learning model for extracting the customer intention from the text features of SEM ads. As we show in Figure 3, for a given ad *A* and its tokenized feature *T*_*A*_, the model will consecutively go through an initial embedding layer, 3 transformer layers, a dense pooling layer, and two feedforward layers before generating the final 512-dimension *normalized* output vector.

#### 3.1.4. Training data

The data we use for training the representation learning model is the search_term_report from the search engine, which provides the historical statistics of interactions (e.g., clicks, impressions) between SEM ads and their relevant search queries. Specifically, for each SEM ad, we will extract historical click numbers between the ad and each search query that leads to the clicks during a given time window. Together with interactive metric *I* defined in Equation 5, we create a data-set D containing all the tuples of SEM ads having co-clicked queries together with their interactive metric. In addition to the above positive instances, we need negative instances to cover larger support of the distribution. For that purpose, we sample a certain number of ad tuples without co-clicked queries and append the tuples onto the data-set D by assigning them with an interactive metric value of −1. The steps of constructing training data are illustrated in the left part of Figure 3. For the best practice, the ratio between positive tuples and negative tuples should be approximately equal to the average positive interactive metric in the feedback data.

#### 3.1.5. Model training

Let *f*_θ_(·) denote a customer intention model with parameter vector θ. Given an ad tuple (*A*_*i*_, *A*_*j*_) along with their interactive metric *I*_*ij*_, we define the loss function as


(6) $$\begin{array}{l} -I_{ij}\log\sigma \left(f_{\theta }{\left(T_{A_{i}}\right)}^{T}f_{\theta }\left(T_{A_{j}}\right)\right) \end{array}$$

Where σ(·) is the sigmoid function. The inner product of $f_{\theta }{\left(T_{A_{i}}\right)}^{T}f_{\theta }\left(T_{A_{j}}\right)$ captures the cosine similarity between the embeddings of (*A*_*i*_, *A*_*j*_), given that output vectors of the model *f*_θ_(·) are normalized. The structure of the model, together with the procedure for calculating the loss function, are presented on the right side of Figure 3. The optimization problem for finding the optimal θ is now given by:


(7) $$\begin{array}{l} \theta^{⋆}=\underset{\theta \in \Theta }{\text{arg min}}\underset{\left(A_{i},A_{j}\right)\in DT}{\sum }-I_{ij}\log\sigma \left(f_{\theta }{\left(A_{i}\right)}^{T}f_{\theta }\left(A_{j}\right)\right) \end{array}$$

The objective (7) indicates that the larger the interactive metric between two ads, the more impact this ad instance will carry when determining model parameter θ. Including the negative instances will allow the model to further separate ads that lack a shared customer intention. Moreover, using negative samples can avoid over-fitting and the corner case where all SEM ads have a similar embedding. We use the ADAM (Kingma and Ba, 2015) optimizer, a variant of stochastic optimization for training (7).

### 3.2. Multi-stage SEM ads clustering algorithm

In what follows, we discuss clustering with ads embedding. Due to the high volume of ads in modern SEM, though many efficient machine learn models have been introduced (Hartigan and Wong, 1979; Sakshi et al., 2015; Schubert et al., 2017), it is still impractical to apply the clustering algorithms that require computing all the pair-wise distances. Here, we present a multi-stage method that leverages the SEM ads' taxonomy and significantly reduces the computation demand.

#### 3.2.1. SEM ads classification

The first step of the multi-stage clustering algorithm is to classify each SEM ad into one of the *product types*, which can be any taxonomy that is labeled for the items: electronics, beverage, etc. Most companies have a predefined taxonomy for each item, which should be actively exploited. SEM ad with only one item can be directly concluded to its product type, and serve as the training sample of the taxonomy classification model. For SEM ads with more than one item, we train a feedforward neural network to predict each ads' product type, which takes the embedding of the SEM ad as input.

#### 3.2.2. Clustering within each product type

Following the classification, we apply the “bottom-up” Agglomerative clustering (Joe, 1963) using embedding vectors as features to create mutually exclusive ad groups for the SEM ads within each product type. Naturally, the cosine distance is employed as the *linkage metric* and it also allows us to determine the threshold based on which the final clusters are formed. We point out that the first classification step significantly reduces the computation complexity compared with directly clustering all the ads.

## 4. Predicting RPC for SEM ads

In the next step, we build a machine learning model for each ads cluster to predict the key quantity of *RPC*_*g*_, i.e., the revenue per click, whose role was illustrated in Section 2.2.

### 4.1. Features

The features we use for predicting *RPC* can be categorized into three classes: 1. the historical feedback statistics such as clicks and conversions; 2. the activity metric for the ad's landing pages such as bounce rate and number of visits; 3. contextual features of the ad, which are currently the average of ad intention embedding vectors for each ad group. Our real world application experience suggests that when sufficient, historical feedback data are the most important features in forecasting future *RPC*s, whereas the sites and ads contextual features are generally serving as complementary roles in prediction when the ad groups have relatively scarce feedback data.

### 4.2. Model selection

There are varieties of machine learning models available for predicting *RPC*. On the high level, the predictive models can be categorized into two frameworks: 1. Typical regression models where historical feedback data together with the contextual embedding vectors of ads are features joint. 2. Time series sequence models which leverage the time series structure of the feedback data to predict *RPC*.

**Figure 4:**
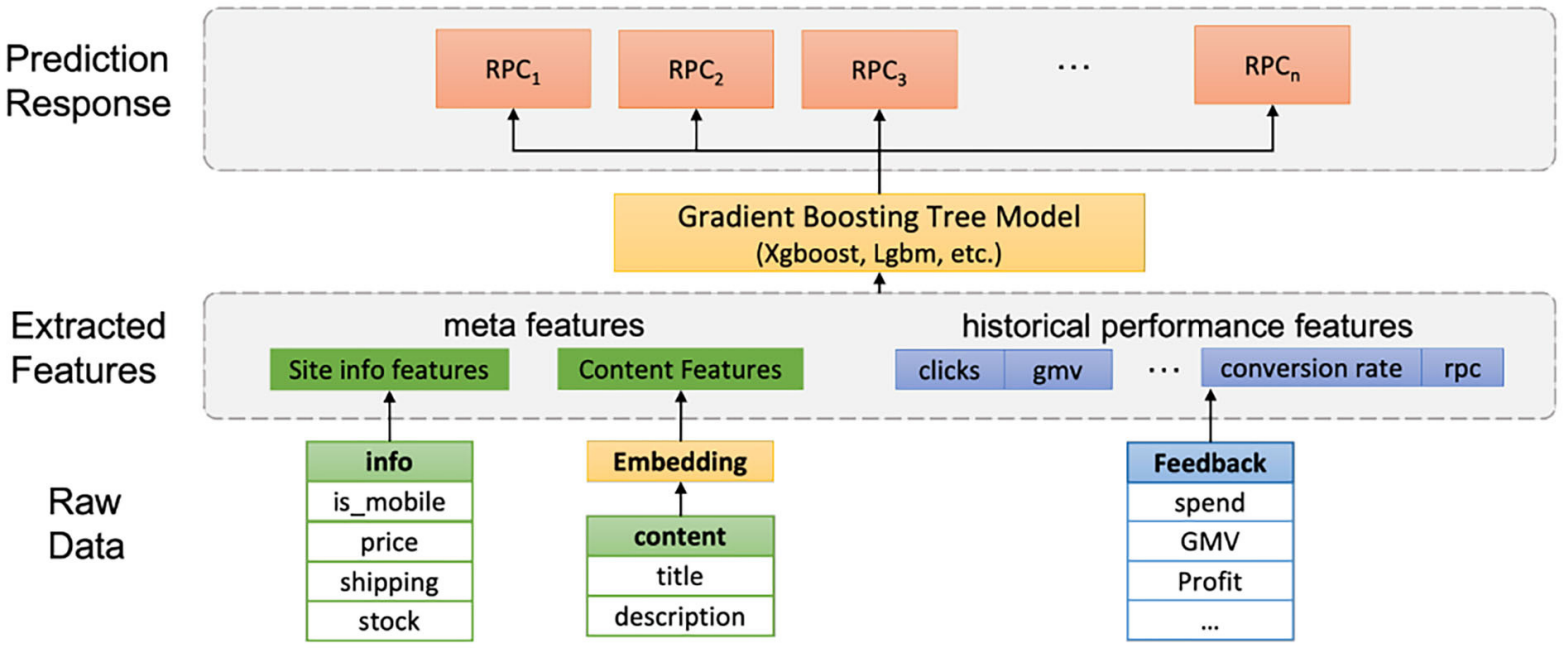
Model training: Gradient boosting regression trees.

For typical regression models, the gradient boosting regression tree (GBRT) usually stands out since it excels at attaining high prediction accuracy on tabular data. Recently, the development of high efficiency packages such as *XGboost* and *LightGBM* (Guolin et al., 2017) makes training an accurate boosting regression tree time manageable on the scale of our SEM ads' features data. The process of training the gradient boosting tree on SEM ads is illustrated in Figure 4.

Intuitively, one would argue that the sequential time series model would outperform the tree regression model. However, the discussions and experiments in Section 5.3 shows that GBRT can achieve similar accuracy metric as complex time series models such as recurrent neural networks (LSTM) with much higher computational efficiency.

### 4.3. Model training

We choose the clicks-weighted square error as the loss function for model training because the ad groups with higher clicks often have more impact on the business. Formally, by denoting the parameter of the model by η∈H and the total clicks of the ad group by *C*_*g*_, the objective function for predicting a reward per click(*RPC*_*i*_) is given by:


(8) $$\begin{array}{l} \eta^{⋆}=\underset{\eta \in H}{\text{arg min}}\frac{\sum_{g\in G}C_{g}{\left(r_{i}^{\eta }\left(X_{g}\right)-RPC_{ig}\right)}^{2}}{\sum_{g\in G}C_{g}}, \end{array}$$

Where *r*_η_ is the RPC predictive model.

## 5. Experiments and numerical analysis

### 5.1. Ablation study

Here, we conduct an ablation study to compare the performance of the ads representation model developed in Section 3 with other candidates for embedding models. To this end, we select a few pre-trained text embedding models as the candidates for comparison. The performance embedding models is determined by the accuracy of the model to correctly predict whether a pair of ads is positively connected or not. Positive connectivity indicates whether the two ads have shared co-clicks, as explained in Section 3.2. The data selected for the evaluation consists of 500 positive and 500 negative pairs. Note that the evaluation data is not used for model training at Section 3.2.

Following the notations in Section 3, for a given ad pair (*A*_*i*_, *A*_*j*_) and a given embedding model *f*, the probability of two ads being positively connected is calculated by the sigmoid transform of the two ads dot-product, expressed as $\sigma \left(f{\left(T_{A_{i}}\right)}^{T}f\left(T_{A_{j}}\right)\right)$. The performances of different models, measured by various accuracy metrics^4^, are displayed in Table 1. The accuracy metrics of different models exemplify that the customer embedding model depicted in Figure 3 performs the best in recognizing the connectivity of the SEM ads.

**Table 1:** Search engine marketing (SEM) ads vs. ad groups: Data-set overview.

**Model**	**AUC**	**F score**	**Accuracy**
Customer intention embedding	90%	86%	87%
Bert pre-trained	84%	82 %	81%
Universal sentence encoder pre-trained	78%	75%	75%
Glove pre-trained	76%	70%	68%

### 5.2. Numerical analysis on bids, clicks, and cost

In this section, we present the real-world numerical evidence to justify the validity of Assumption 2.1.2, which is the key principle of our bidding system. Recall that the main idea of Assumption 2.1.2 implies that the bid price is linearly correlated to both clicks and cost per click (*CPC*). In order to validate the claim, we pick a time window of a month and select all the SEM ads with clicks larger than 0 during the time window for our numerical analysis.

Following the protocol of cluster-based bidding, we organize the SEM ads into a set of ad groups G and retrieve the monthly statistics for each adgroup. We fit a regression model of bid value *b*_*g*_ against *CPC*_*g*_, i.e, *CPC*_*g*_ = β_0_+β_1_ · *b*_*g*_. The parameters of the linear model β_0_ and β_1_ are determined through clicks weighted mean square error. Results of the linear model, including goodness-of-fit measures, are presented in Table 2.

**Table 2:** Linear regression: *CPC*_*g*_ ~ β_0_ + β_1_ · *b*_*g*_.

**Coefficients**	**Estimation**	***t*-value**	***p*-value**
β_0_(intercept)	0.07	–1.34	0.13
β_1_(*CPC*_*g*_)	0.84	4.45	< 0.01
Multiple R-squared: 0.931, Adjusted R-squared: 0.94			
F-statistics: 241.5, *P* < 0.01			

**Figure 5:**
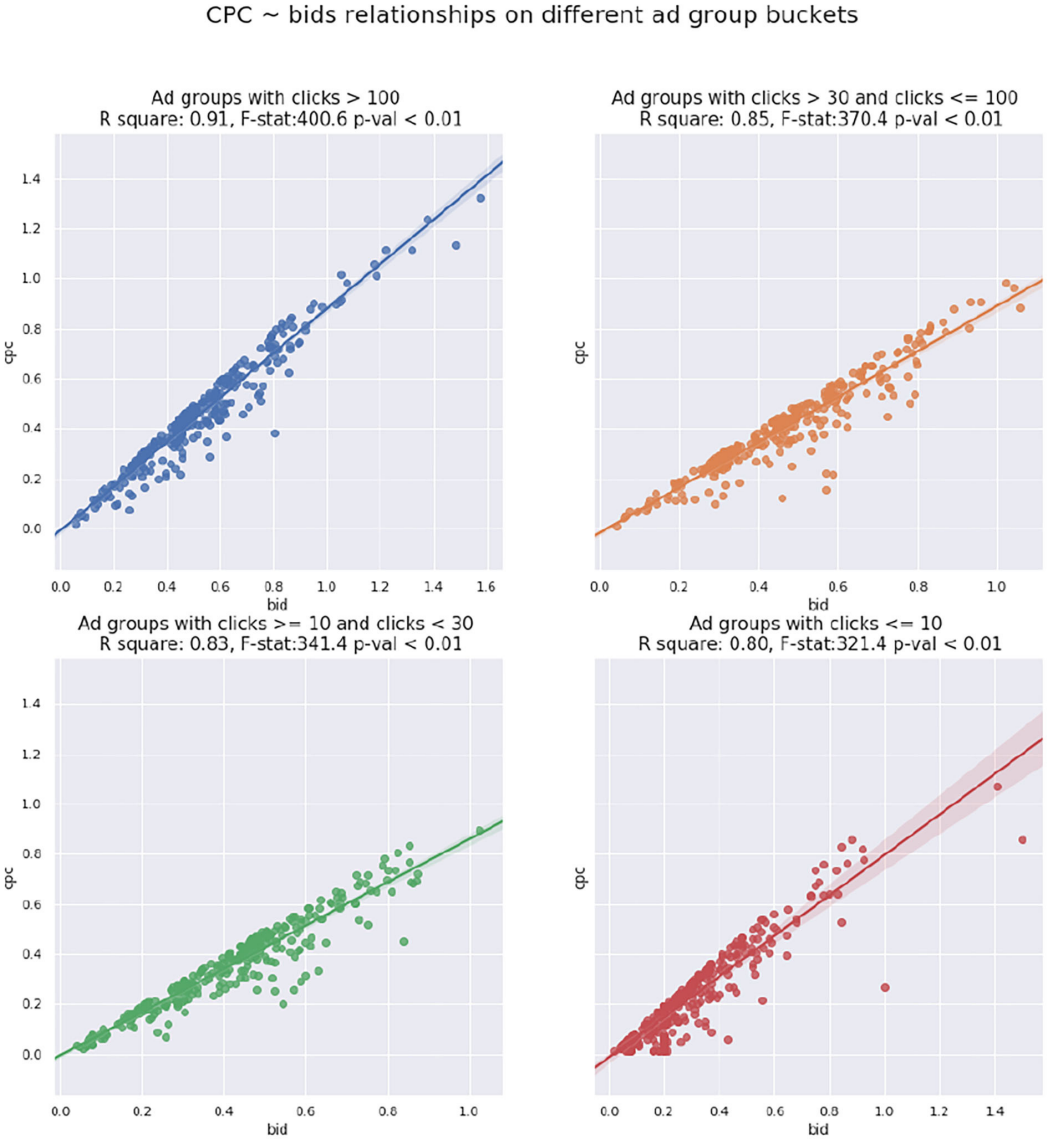
Cost per click (CPC) bids scatter plot with regression line for different ad group buckets regarding clicks.

The significance of the slope, together with the high R-squared value in Table 2 indicates a statistically significant linear relationship between the bidding value *b*_*g*_ and corresponding *CPC*_*g*_. Also notice that the slope β_1_ here is close to 1. Furthermore, we split our ad groups into different buckets according to their monthly clicks, and Figure 5 reveals the scatter plot and regression line between bid and *CPC* of sample ad groups among different buckets. The results in Figure 5 suggest that the higher clicks an ad group has, the stronger the linear relationship between its bid value and *CPC*.

**Figure 6:**
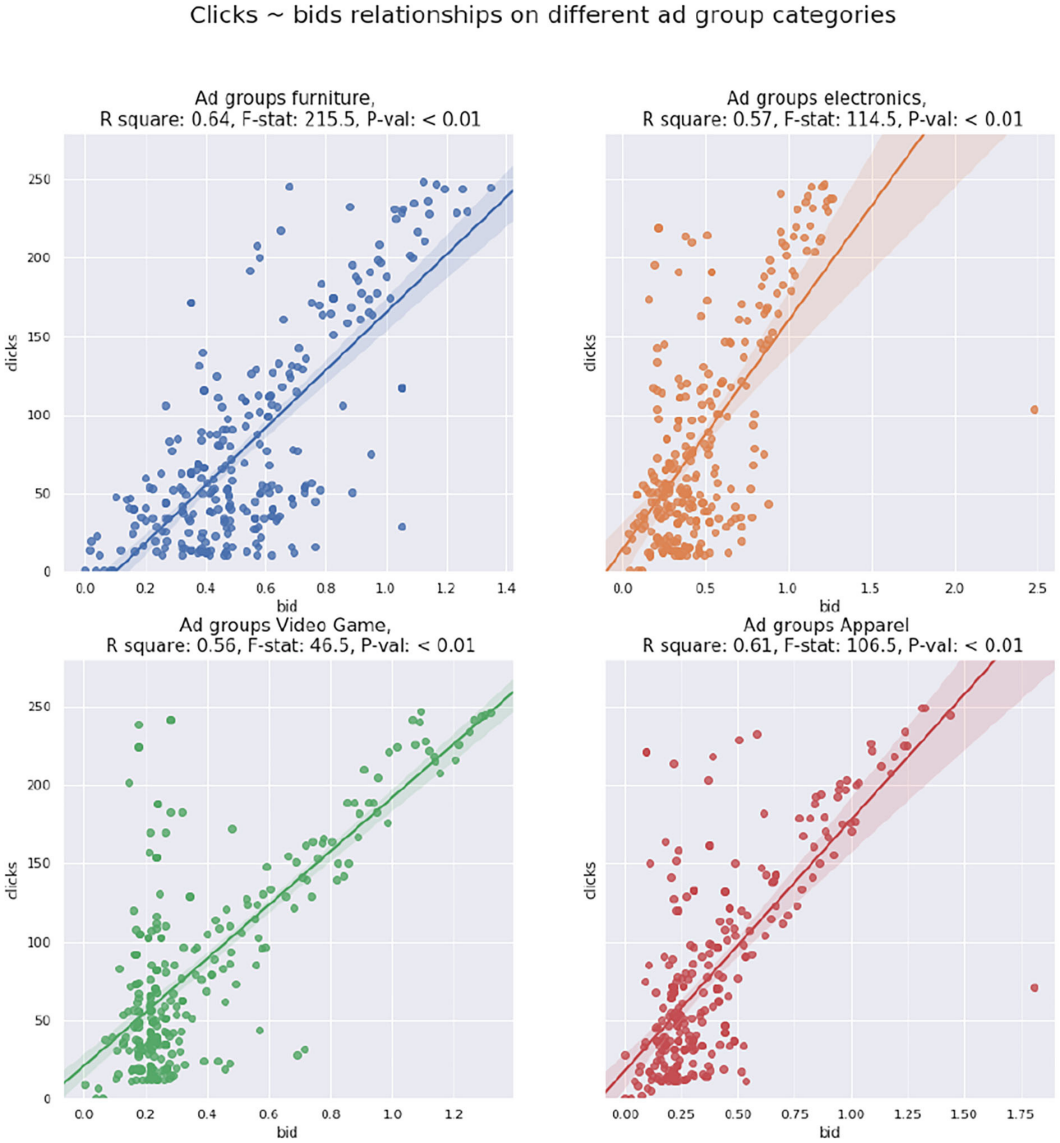
Clicks bids scatter plot with regression line for product types of ad groups.

Similarly, we fit another regression line of *b*_*g*_ against clicks *C*_*g*_ of ad group *g*. According to Table 3, the two metrics are also statistically correlated. Moreover, we fit regressions lines separately on different product types of ad groups based on Section 3.2. As revealed in Figure 6, clicks and bid values are correlated across different product types, and such correlations are usually stronger than the one derived from the entire dataset, according to the goodness-of-fit measures.

**Table 3:** Linear regression: *C*_*g*_ ~ β_0_ + β_1_ · *b*_*g*_.

**Coefficients**	**Estimation**	***t*-value**	***p*-value**
β_0_(intercept)	2.1	1.56	0.09
β_1_(*CPC*_*g*_)	104.6	2.97	< 0.02
Multiple R-squared: 0.59, Adjusted R-squared: 0.6			
F-statistics: 150.5, *P* < 0.01			

### 5.3. Experimental study

We conducted both offline and online experiments to answer the following questions:

**Q1:** Can ads clustering improve the RPCs prediction accuracy for different types of rewards by addressing the spareness of feedback data?

**Q2:** Does the proposed two-step framework improve the business performance?

#### 5.3.1. Offline experiment: Prediction accuracy comparison

The offline experiment is designed to test whether the proposed clustering methods address the sparseness issue and improve the prediction accuracy of RPCs of different rewards. Here, we choose gross merchandise value (GMV) and commercial profits (CP) as the rewards for our experiment due to their close ties with our needs. Moreover, we select a set of ads with a total number of ~21 million, and compare the accuracy of RPC predictions of 1. directly applying RPC predictions on each SEM ad (the baseline singular-ad-based algorithm); 2. clustering SEM ads before predicting RPC for each ad cluster (our cluster-based bidding algorithm). For a fair comparison, we evaluate the performance metric based on each ad and set the predicted RPC of each ad equivalent to the predicted RPC of its belonging ad cluster when using the second approach. According to the operation protocol of Walmart, we predict the weekly RPCs of both GMV and CP as described in Section 4. For the proposed approach 2, we apply the methods introduced in Section 3 to cluster SEM ads into ad groups, and aggregate the ad features within each ad group. The summary statistics for the ad groups and the original SEM ads are displayed in Table 4. Notice that the experiment has been redone since the publication of Cheng et al. (2021), therefore both the data and samples are slightly different from the results in Cheng et al. (2021).

**Table 4:** Search engine marketing ads vs. ad groups: Data-set overview.

	**SEM ads**	**SEM ad groups**
Dataset sample size	21.4 M	1.9M
Missing feature (proportion)	89.7%	52.2%
Non-empty response ratio	6.8 %	37.4 %
Relative response *RPC*_*G*_ variance	100%	53%
Relative response *RPC*_*C*_ variance	100%	60%

#### 5.3.2. Notation remark

Note that RPCs of GMV and CP can also be interpreted as GMV per click and CP per click, respectively. For notation convenience, in the following, we use *RPC*_*G*_ and *RPC*_*C*_ to denote the corresponding RPC of GMV and CP.

In Table 4, the proportion of feature missingness is calculated based on the non-contextual features, and due to Walmart's privacy policy, the variances of the *RPC*_*G*_ and *RPC*_*C*_ response variables are presented as percentage proportions to the largest among the two datasets. Table 4 manifests the two benefits of ads clustering: 1. the feature sparseness is dramatically improved as exemplified by the reduced missing feature proportion, 2. the reduced variance of the response variable indicates that the clustering algorithm tends to produce a more robust output for the downstream *RPC* modeling.

**Figure 7:**
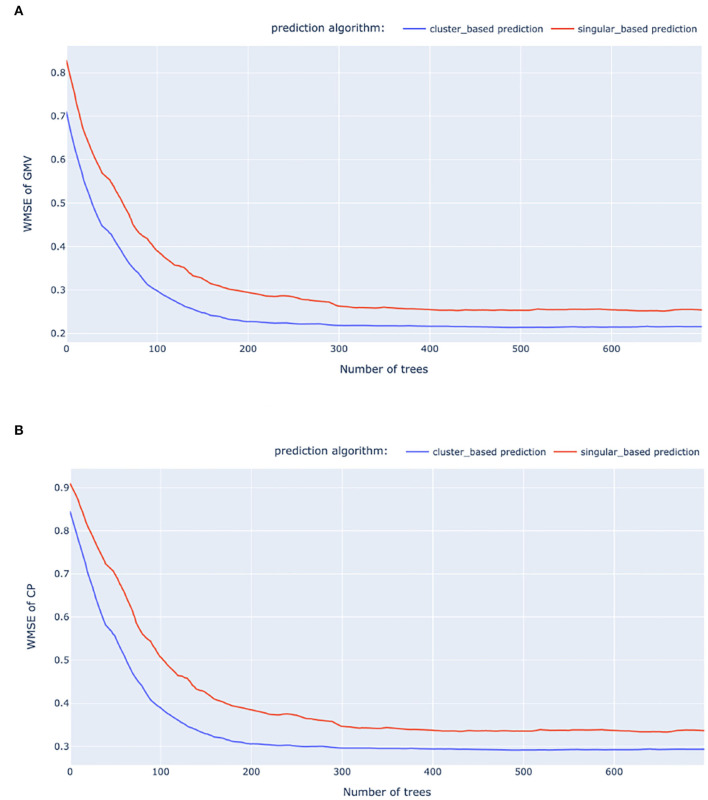
Relative WMSE of the baseline and our approach when using GBRT. **(A)** GMV WMSE. **(B)** CP WMSE.

We experimented with three machine learning models for predicting the weekly *RPC*_*G*_ and *RPC*_*C*_: *linear regression* (LR) model, *LSTM*, and *gradient boosting regression tree* (GBRT). For building *LSTM* models, we re-construct the feature dataset to time series sequences. We split the dataset into training, validation, and test by 80% − 10% − 10%, where the test dataset is used to report the predictive accuracy of the trained models. In addition to the click-weighted MSE (WMSE) mentioned in Section 4, we also include the click-weighted MAE (WMAE) as the performance metric. The performances of the trained models are displayed in Table 5. Figure 7 presents examples of the gradient boosting trees on *RPC*_*G*_ and *RPC*_*C*_ predictions when applied to the baseline and our approach, under their best hyper-parameter combinations. Due to the privacy policy, we provide the accuracy metric with respect to the baseline model, which is Linear regression (LR) on the singular-ad-based algorithm. The model training, including hyper-parameter tuning, is conducted on a Linux system with 256 core 2.80GHz CPUs and 1,600 GB memory.

**Table 5:** The *RPC*_*G*_ and *RPC*_*C*_ predictions accuracy (relative to LR on singular-ad setting), and the offline model training time.

**Metric**	**Predictive model**	**WMSE (Relative to LR Singular)**		**WMAE (Relative to LR Singular)**		**Training time**	
		**Singular based**	**Cluster based**	**Singular based**	**Cluster based**	**Singular based**	**Cluster based**
*RPC*_*G*_	LR(reference point)	100%	92%	100%	86%	8m	2m
	RNN	24%	20%	30%	24%	70h	22h
	Gradient Boosting	25%	21%	29%	23%	4.5h	1h
*RPC*_*C*_	LR(reference point)	100%	88%	100%	75%	7m	2m
	RNN	29%	22%	28%	20%	66h	20h
	Gradient Boosting	30%	23%	28%	21%	4.2h	1h

The results from Figure 7 and Table 5 suggest that RPC predictions *via* ad clustering consistently achieve better performances compared with the singular ad prediction, both in the cases of GMV and CP. Furthermore, the computational time for training RPC at the cluster level is considerably less than the singular-ad level. Given its high accuracy and computational efficiency, gradient boosting regression trees (GBRT) emerges as a practical choice for real world application of RPC predictions. We would point out that the training time presented in Table 5 does not include the train data preparation time, which puts further disadvantages on sequential models.

#### 5.3.3. Online experiment: Business efficiency comparison

We designed the online AB testing experiment to see whether the clustering-based bidding improves business performance, mainly reflected by the weighted sum of rewards under budget. Following the offline experiment section, we select GMV and CP as the primary rewards and test our proposed algorithm on three pairs of weights: (GMV 0.7, CP 0.3), (GMV 0.5, CP 0.5), and (GMV 0.3, CP 0.7). To this end, we use stratified sampling to select three groups of SEM ads as our target ads pool. Furthermore, we compare the business performances between clustering-based bidding and traditional singular-ad-based bidding algorithms when applied to the selected SEM ads. Each group of SEM ads is optimized toward one selected corresponding pair of weighted rewards, as shown in Table 6.

**Table 6:** Results for 3 online AB tests regarding 3 different reward weights.

	**Group 1**		**Group 2**		**Group 3**	
**Reward weights**	**GMV: 0.7, CP: 0.3**		**GMV: 0.5, CP: 0.5**		**GMV: 0.3, CP: 0.7**	
Control/Test	Control	Test	Control	Test	Control	Test
Spend	100%	101%	100%	98%	100%	97%
GMV	100%	124%	100%	104%	100%	75%
CP	100%	85%	100%	107%	100%	130%
Weighted Rewards	100%	105%	100%	106%	100%	105%
*t*-test Statistics	2.7, *p*-value: 0.02		2.8, *p*-value: 0.01		2.1, *p*-value: 0.03	

Here, we leverage the Draft & Experiment platform from Google Adwords^5^ to create three pairs of control and test campaigns that host each group of 200k ads by duplicating the original ads into two copies. For each pair of campaigns, the singular-based and clustering-based bidding algorithms are applied to the control and test campaign, respectively.

In Google Draft & Experiment setting, the control and test campaigns start simultaneously, and during the test, Google evenly splits incoming traffic to ensure a fair comparison. For each pair of control and test campaigns, The experiment session consists of 1 week of AA test and three following weeks of AB test, during which the control and test campaigns are treated differently. The results of 3 online AB tests corresponding to the three pairs of reward weights are presented in Table 6, where we present metrics relative to the control campaign.

Table 6 exemplifies that the cluster-based algorithm outperforms the singular-based bidding algorithm across all sets of reward weights, according to the weighted sum of rewards and spend level between control and test campaigns. Furthermore, notice that when the bidding algorithm is designed toward optimizing the blended business objectives, it will not necessarily optimize each of the objectives.

To further justify our conclusion, for each of the AB tests, we perform a paired *t-test* on the weighted RPS of two campaigns, which are shown at the bottom of Table 6.

## 6. Conclusion and future study

This article introduces a two-step clustering-based SEM bidding system that integrates modern representation learning with the Transformer language model. We describe the detailed development infrastructure of the multi-objective bidding system that may bring insights to both practitioners and researchers in this domain. The offline and online experiments show that the proposed system compares favorably to the alternatives in terms of accuracy and training efficiency. Our successful deployment of Walmart e-commerce further reveals combining clustering with a modern representation learning as a scalable solution for industrial bidding systems. In the future, the clustering and SEM ads embedding models can be extended by adding pixel features of the items, and we can incorporate more complex reward-spend functions into our bidding systems.

## Data availability statement

The original contributions presented in the study are included in the article/supplementary material, further inquiries can be directed to the corresponding author.

## Author contributions

CJ is the main author for initializing the paperwork and contributed to most parts of the paper. ZW mainly contributed to the keywords clustering section and experiment section. DX helped with paper writing and modification. WS mainly supervised the paper writing and the related project. All authors contributed to the article and approved the submitted version.

## Conflict of interest

Authors CJ, ZW, DX, and WS were employed by company Walmart Labs.

## Publisher's note

All claims expressed in this article are solely those of the authors and do not necessarily represent those of their affiliated organizations, or those of the publisher, the editors and the reviewers. Any product that may be evaluated in this article, or claim that may be made by its manufacturer, is not guaranteed or endorsed by the publisher.

## Footnotes

^1^An original version of the article can be found in the KDD IRS workshop (Cheng et al., 2021). Compared to the workshop article, we extend the bidding optimization model from single objective to multi-objective, add more discussions on the predictive model building Section 4 and include more extensive numerical results and analysis in the experiments Section 5.

^2^First price auction in sponsored search is referred to the mechanism that once a click occurs, the bidder will be charged with the bidding-price it provides for the ad slot of the search result (Turocy et al., 2007).

^3^Second price auction is referred to the rule that once an ad is clicked, the related advertiser will be charged with the highest bid to participate in the auction that is lower than the bid value the advertiser is offering (Benjamin et al., 2005).

^4^F score and accuracy are calculated according to the best possible probability thresholds.

^5^https://ads.google.com, where the max capacity for a campaign is 200,000.
